# Draft Genome Sequences of *Elizabethkingia meningoseptica*

**DOI:** 10.1128/genomeA.00444-13

**Published:** 2013-07-11

**Authors:** Stephanie A. Matyi, Peter R. Hoyt, Akira Hosoyama, Atsushi Yamazoe, Nobuyuki Fujita, John E. Gustafson

**Affiliations:** Department of Biochemistry and Molecular Biology, Oklahoma State University, Stillwater, Oklahoma, USAa; Biological Resource Center, National Institute of Technology and Evaluation, Tokyo, Japanb

## Abstract

*Elizabethkingia meningoseptica* is ubiquitous in nature, exhibits a multiple-antibiotic resistance phenotype, and causes rare opportunistic infections. We now report two draft genome sequences of *E. meningoseptica* type strains that were sequenced independently in two laboratories.

## GENOME ANNOUNCEMENT

The genus *Elizabethkingia* was derived in 2005 following a series of systematic investigations that led to the reclassification of members previously found within the genera *Flavobacterium* and *Chryseobacterium* (1–4). Currently, *Elizabethkingia* is represented by the three species *Elizabethkingia miricola* (5), *Elizabethkingia meningoseptica* (6, 7), and *Elizabethkingia anophelis* (8).

*E. meningoseptica* expresses a multiple-antibiotic resistance phenotype and causes infections primarily within immunocompromised individuals (9–11). The type strain of *E. meningoseptica* was isolated in 1958 in a case of neonatal meningitis (6, 7). We now report the draft genome sequences of two *E. meningoseptica* type strains, NBRC 12535^T^ and ATCC 13253^T^. Both of these culture collection strains are representatives of the original *E. meningoseptica* strain isolated by Elizabeth King and colleagues (6).

The draft genome sequences of NBRC 12535^T^ and ATCC 13253^T^ were prepared at the National Institute of Technology and Evaluation, Tokyo, Japan, using Illumina HiSeq 1000 technology and at Oklahoma State University using the Roche 454 GS Junior platform, respectively. Genomic DNA to be sequenced was isolated from overnight cultures (30°C) of NBRC 12535^T^ grown on nutrient agar containing 75% artificial seawater and ATCC 13253^T^ grown in nutrient broth. Sequencing of NBRC 12535^T^ generated 3,974,452 reads (103× coverage; average read length, 99.4 bp) that were assembled with the Newbler assembler (v2.6). ATCC 13253^T^ sequencing produced 223,447 reads (29.7× coverage; average read length, 504.9 bp) that were assembled with the Roche GS *de novo* assembler (v2.7). Both draft genome sequences were uploaded to the Rapid Annotations using Subsystems Technology (RAST) server for annotation (12).

The NBRC 12535^T^ draft genome sequence is 3,840,286 bp in length (36.2% G+C content) and includes 3,519 protein-coding regions distributed in 34 contigs (>500 bp). The ATCC 13253^T^ draft genome sequence is 3,797,222 bp (35.2% G+C content) in length and includes 3,486 protein-coding regions distributed in 115 contigs (>200 bp). One hundred eleven contigs (representing 3,795,245 bp) of the ATCC 13253^T^ sequence demonstrated 99 to 100% nucleotide identity with the 34 contigs of the NBRC 12535^T^ sequence, which indicates that these draft genome sequences are essentially the same.

The nucleotide alignment of several highly conserved genes from the *E. meningoseptica* draft genome sequences and *E. anophelis* R26^T^ (gene and nucleotide identities are as follows: *gln*, 86%; *gyrB*, 87%; *recA*, 88%; *atpD*, 92%; and *dnaK*, 92%) strongly supports previous findings that *E. anophelis* is at least a separate species (8). The 16S rRNA sequences of these two species are 98% identical, which is not very definitive for speciation (13). *E. meningoseptica* is resistant to β-lactam antibiotics due to the production of metallo-β-lactamases (MBLs) and extended-spectrum β-lactamases (ESBLs) (14–16). Two MBL variants (*bla*_GOB-17_ and *blaB3*) and one ESBL gene (*blaA*_CME-1_) were found in both *E. meningoseptica* draft genome sequences and were aligned with similar *E. anophelis* genes. A comparison of the β-lactamase orthologs from these two species revealed only 74% to 85% amino acid identity. This finding confirms that *Elizabethkingia* species, which are ubiquitous in nature (17), may act as potential reservoirs of novel β-lactamase genes.

### Nucleotide sequence accession numbers.

These whole-genome shotgun projects have been deposited at DDBJ/EMBL/GenBank under the accession no. BARD00000000 for NBRC 12535^T^ and ASAN00000000 for ATCC 13253^T^.

## References

[1] Ursing J Bruun B 1987 Genetic heterogeneity of *Flavobacterium meningosepticum* demonstrated by DNA-DNA hybridization. Acta Pathol. Microbiol. Immunol. Scand. B 95:33–39 3565016 10.1111/j.1699-0463.1987.tb03084.x

[2] Bruun B Ursing J 1987 Phenotypic characterization of *Flavobacterium meningosepticum* strains identified by DNA-DNA hybridization. Acta Pathol. Microbiol. Immunol. Scand. B 95:41–47 3565017 10.1111/j.1699-0463.1987.tb03085.x

[3] Vandamme P Bernardet J-F Segers P Kersters K Holmes B 1994 New perspectives in the classification of the *Flavobacteria*: description of *Chryseobacterium* gen. nov., *Bergeyella* gen. nov., and *Empedobacter* nom. rev. Int. J. Syst. Bacteriol. 44:827–831

[4] Kim KK Kim MK Lim JH Park HY Lee ST 2005 Transfer of *Chryseobacterium meningosepticum* and *Chryseobacterium miricola* to *Elizabethkingia* gen. nov. as *Elizabethkingia meningoseptica* comb. nov. and *Elizabethkingia miricola* comb. nov. Int. J. Syst. Evol. Microbiol. 55:1287–1293 15879269 10.1099/ijs.0.63541-0

[5] Li Y Kawamura Y Fujiwara N Naka T Liu H Huang X Kobayashi K Ezaki T 2003 Chryseobacterium miricola sp. nov., a novel species isolated from condensation water of space station Mir. Syst. Appl. Microbiol. 26:523–528 14666980 10.1078/072320203770865828

[6] Brody JA Moore H King EO 1958 Meningitis caused by an unclassified Gram-negative bacterium in newborn infants. AMA J. Dis. Child. 96:1–5 13544725 10.1001/archpedi.1958.02060060003001

[7] King EO 1959 Studies on a group of previously unclassified bacteria associated with meningitis in infants. Am. J. Clin. Pathol. 31:241–247 13637033 10.1093/ajcp/31.3.241

[8] Kämpfer P Matthews H Glaeser SP Martin K Lodders N Faye I 2011 Elizabethkingia anophelis sp. nov., isolated from the midgut of the mosquito Anopheles gambiae. Int. J. Syst. Evol. Microbiol. 61:2670–2675 21169462 10.1099/ijs.0.026393-0

[9] Yang YS Chun JW Koh JW 2013 Keratitis with *Elizabethkingia meningoseptica* occurring after contact lens wear: a case report. Korean J. Ophthalmol. 27:133–136 23543626 10.3341/kjo.2013.27.2.133 PMC3596618

[10] Issack MI Neetoo Y 2011 An outbreak of *Elizabethkingia meningoseptica* neonatal meningitis in Mauritius. J. Infect. Dev. Ctries. 5:834–839 22169781 10.3855/jidc.1885

[11] Cartwright EJ Prabhu RM Zinderman CE Schobert WE Jensen B Noble-Wang J Church K Welsh C Kuehnert M Burke TL Srinivasan A 2010 Transmission of *Elizabethkingia meningoseptica* (formerly *Chryseobacterium meningosepticum*) to tissue-allograft recipients: a report of two cases. J. Bone Joint Surg. Am. 92:1501–1506 20516326 10.2106/JBJS.I.00502

[12] Aziz RK Bartels A Best AA DeJongh M Disz T Edwards RA Formsma K Gerdes S Glass EM Kubal M Meyer F Olsen GJ Olson R Osterman AL Overbeek RA McNeil LK Paarmann D Paczian T Parrello B Pusch GD Reich C Stevens R Vassieva O Vonstein V Wilke A Zagnitko O 2008 The RAST server: rapid annotations using subsystems technology. BMC Genomics 9:75. 10.1186/1471-2164-9-75 18261238 PMC2265698

[13] Gevers D Cohan FM Lawrence JG Spratt BG Coenye T Feil EJ Stackerbrandt E Van de Peer Y Vandamme P Thompson FL Swings J 2005 Re-evaluating prokaryotic species. Nat. Rev. Microbiol. 3:733–739 16138101 10.1038/nrmicro1236

[14] Yum JH Lee EY Hur S-H Jeong SH Lee HL Yong D Chong Y Lee E-W Nordmann P Lee K 2010 Genetic diversity of chromosomal metallo-β-lactamase genes in clinical isolates of *Elizabethkingia meningoseptica* from Korea. J. Microbiol. 48:358–364 20571954 10.1007/s12275-010-9308-5

[15] Woodford N Palepou M-F Babini GS Holmes B Livermore DM 2000 Carbapenemases of *Chryseobacterium* (*Flavobacterium*) *meningosepticum*: distribution of *blaB* and characterization of a novel metallo-β-lactamase gene, *blaB3*, in the type strain, NCTC 10016. Antimicrob. Agents Chemother. 44:1448–1452 10817691 10.1128/aac.44.6.1448-1452.2000 PMC89895

[16] Rossolini GM Franceschini N Lauretti L Caravelli B Riccio ML Galleni M Frère JM Amicosante G 1999 Cloning of a *Chryseobacterium* (*Flavobacterium*) *meningosepticum* chromosomal gene (*blaA*_CME_) encoding an extended-spectrum class A beta-lactamase related to the Bacteroides cephalosporinases and the VEB-1 and PER beta-lactamases. Antimicrob. Agents Chemother. 43:2193–2199 10471563 10.1128/aac.43.9.2193 PMC89445

[17] Bernardet JF Hugo C Bruun B 2006 The genera *Chryseobacterium* and *Elizabethkingia*. Prokaryotes 7:638–676

